# Protein kinase Cγ negatively regulates the intrinsic excitability in zebrin-negative cerebellar Purkinje cells

**DOI:** 10.3389/fncel.2024.1349878

**Published:** 2024-02-16

**Authors:** Masashi Watanave, Mika Kawachi, Ayumu Konno, Ryo Aoki, Yuuki Fukai, Yasunori Matsuzaki, Ryosuke Kaneko, Hirokazu Hirai

**Affiliations:** ^1^Department of Neurophysiology and Neural Repair, Gunma University Graduate School of Medicine, Maebashi, Japan; ^2^Viral Vector Core, Gunma University, Initiative for Advanced Research, Maebashi, Japan; ^3^KOKORO-Biology Group, Neuroscience Laboratories, Graduate School of Frontier Biosciences, Osaka University, Osaka, Japan

**Keywords:** protein kinase, cerebellum, action potential, Purkinje cells, aldolase C, zebrin, heterogeneity

## Abstract

Protein kinase C γ (PKCγ), a neuronal isoform present exclusively in the central nervous system, is most abundantly expressed in cerebellar Purkinje cells (PCs). Targeted deletion of PKCγ causes a climbing fiber synapse elimination in developing PCs and motor deficit. However, physiological roles of PKCγ in adult mouse PCs are little understood. In this study, we aimed to unravel the roles of PKCγ in mature mouse PCs by deleting PKCγ from adult mouse PCs of PKCγ*^fl/fl^* mice via cerebellar injection of adeno-associated virus (AAV) vectors expressing Cre recombinase under the control of the PC-specific L7-6 promoter. Whole cell patch-clamp recording of PCs showed higher intrinsic excitability in PCs virally lacking PKCγ [PKCγ-conditional knockout (PKCγ-cKO) PCs] than in wild-type (WT) mouse PCs in the zebrin-negative module, but not in the zebrin-positive module. AAV-mediated PKCγ re-expression in PKCγ-deficient mouse PCs in the zebrin-negative module restored the enhanced intrinsic excitability to a level comparable to that of wild-type mouse PCs. In parallel with higher intrinsic excitability, we found larger hyperpolarization-activated cyclic nucleotide-gated (HCN) channel currents in PKCγ-cKO PCs located in the zebrin-negative module, compared with those in WT mouse PCs in the same region. However, pharmacological inhibition of the HCN currents did not restore the enhanced intrinsic excitability in PKCγ-cKO PCs in the zebrin-negative module. These results suggested that PKCγ suppresses the intrinsic excitability in zebrin-negative PCs, which is likely independent of the HCN current inhibition.

## 1 Introduction

The classical protein kinase C (PKC) subfamily includes α, βI, βII, and γ isoforms. The γ isoform of PKC (PKCγ) is expressed solely in neurons of the central nervous system, and plays critical roles in brain functions, such as contextual learning, motor coordination, and neuropathic pain (Abeliovich et al., 1993b; Chen et al., 1995; Malmberg et al., 1997).

In the central nervous system, the cerebellar cortex contains the highest amount of PKCγ (Ase et al., 1988), which is expressed exclusively in Purkinje cells (PCs), the sole output neurons from the cerebellar cortex (Saito and Shirai, 2002; Takahashi et al., 2017). PCs receive excitatory synaptic inputs via parallel fibers and a climbing fiber, together with inhibitory inputs from cortical interneurons. These inputs are integrated during dendritic transmission and converted into action potentials, which are transferred along the PC axon to cerebellar nuclei. Generation or absence of spikes in PCs, which is a critical determinant of cortical output, is influenced by intrinsic firing properties of PCs. The intrinsic neuronal excitability is regulated by various membrane channels such as Ca^2+^-dependent K^+^ channels and hyperpolarization-activated cyclic nucleotide-gated (HCN) channels (Belmeguenai et al., 2010; Byczkowicz et al., 2019; Grasselli et al., 2020; Roth and Hu, 2020). However, the modulation is so complicated that the mechanism underlying the intrinsic excitability of PCs has not been fully clarified yet.

PCs are composed of heterogeneous populations with different expression profiles of channel and signaling proteins, resulting in distinct intrinsic firing properties. The representative PC classification is based on the aldolase C (zebrin) expression level. PCs with high zebrin expression levels and those with low zebrin expression levels form the parasagittal stripe in the cerebellum (Brochu et al., 1990). PCs in stripes with different expression levels of zebrin show different properties of intrinsic firing; namely, zebrin-positive (Z+) PCs exhibit lower firing frequency than zebrin-negative (Z−) PCs (Zhou et al., 2014; Viet et al., 2022).

The differences in zebrin expression levels in PCs located in different striped regions are in parallel with the differences in expression levels of other proteins; for example, Z+ PCs abundantly express glutamate transporter EAAT4 and phospholipase beta-3 (PLCβ3), while Z− PCs richly express the b-type splicing variant of metabotropic glutamate receptor 1 (mGluR1b), transient receptor potential C3 type (TRPC3), and PLCβ4 (Cerminara et al., 2015; Wu et al., 2019). Notably, the molecules heterogeneously expressed in Z+ and Z− PCs, such as mGluR1b, PLCβ, and TRPC3, are involved in a PKC signaling pathway. In PCs, glutamate binding to Gq/11 protein-coupled mGluR1 activates PLC, which produces diacylglycerol and inositol-triphosphate. Diacylglycerol and inositol-triphosphate-induced cytoplasmic calcium elevation activates PKC (Kano et al., 2008; Hirai and Kano, 2018). Although physiological roles of PKCγ in adult mouse PCs are little understood, differences in the molecules involved in PKC activation may influence the strength of PKC activation in Z+ and Z− PCs, which may account for the differences in their intrinsic firing properties. In this study, we explored the mechanism underlying the higher intrinsic excitability of Z− PCs in terms of PKCγ involvement.

## 2 Materials and methods

### 2.1 Animals

PKCγ-deficient mice (Abeliovich et al., 1993a) were provided by Dr. Masanobu Kano (University of Tokyo, Japan). The PKCγ*^fl/fl^* mice were generated in our previous study (Watanave et al., 2022). All mice used in this study were maintained on a C57BL/6J genetic background in our breeding colony at the Institute of Experimental Animal Research, Gunma University Graduate of Medicine, Gunma, Japan. Homozygous PKCγ-deficient and PKCγ*^fl/fl^* mice were obtained by crossing respective fertile heterozygous animals and were genotyped by PCR. RGS8-EGFP mice (Kaneko et al., 2018) were crossed with mice from the PKCγ-deficient or PKCγ*^fl/fl^* mouse lines to obtain mice carrying both genotypes, which was confirmed by PCR. All procedures regarding the care and treatment of animals were carried out according to the Japanese Act on the Welfare and Management of Animals, and the experimental protocol was approved by the Institutional Committee of Gunma University (approval numbers 23-018 and 21-065).

### 2.2 Preparation of adeno-associated virus vectors

We used the expression plasmid (pAAV-L7-6-minCMV-PKCγ-mCherry-WPRE, pAAV-L7-6-minCMV-GFP-P2A-Cre-WPRE or pAAV-L7-6-minCMV-mCherry-P2A-Cre-WPRE) for AAV9 or AAV PHP.eB (Chan et al., 2017) vector production. The AAV vectors were designed to express GFP (or mCherry) and PKCγ (or Cre) under the control of L7 promotor (Sawada et al., 2010; Nitta et al., 2017) with minCMV (Matsuzaki et al., 2014). The woodchuck hepatitis virus posttranscriptional regulatory element (WPRE) sequence was inserted following PKCγ (or Cre).

Recombinant single-strand AAV vectors were produced by transfection of HEK293T cells (Thermo Fisher Scientific, Waltham, MA, USA) with pAAV/L7-minCMV- PKCγ-mCherry-WPRE (or pAAV-L7-6-GFP-P2A-Cre-WPRE or pAAV-L7-6-mCherry-P2A-Cre-WPRE), pAAV2/9 (provided by Dr. J. Wilson; or pAAV-PHP.eB), and a helper plasmid (Stratagene, La Jolla, CA, USA) using an ultracentrifuge purification method described previously (Konno and Hirai, 2020). The genomic titers of the purified AAV vectors were determined by quantitative real-time PCR using Power SYBR Green Master Mix (Thermo Fisher), according to the manufacturer’s instructions, with the primers 5’-CTGTTGGGCACTGACAATTC-3’ and 5’-GAAGGGACGTAGCAGAAGGA-3’ targeting the WPRE sequence. Expression plasmid vectors were used as standards.

To eliminate PKCγ expression from mature PKCγ*^fl/fl^* mouse PCs, AAV9 vectors expressing Cre recombinase together with GFP or mCherry under the PC-specific L7-6 promoter [10 μl, 1.0 × 10^11^ vector genomes (vg) /ml] were injected into the cerebellum of 3–5-weeks old PKCγ*^fl/fl^* mice. For the PKCγ rescue experiment, intravenous injection of the PC-targeting AAV PHP.eB vectors expressing mCherry PKCγ (100 μl, 5.0 × 10^12^ vg/ml) was administered to 3-week-old systemic PKCγ-null mice [postnatal day (P)21–P25].

### 2.3 Cerebellar and intravenous injections

For direct cerebellar injection of AAV9 vectors, mice were deeply anesthetized with a combination of ketamine (100 mg/kg body weight) and xylazine (10 mg/kg body weight), and placed in a stereotactic frame. The skin covering the occipital bone was cut, and a burr hole was created 2 mm caudal from the lambda. The tip of a Hamilton syringe (33 gauge) (Hamilton Company, Reno, ND, USA) with an attached micropump (UltraMicroPump II; World Precision Instrument, Sarasota, FL, USA) was inserted 1.6 mm below the pia mater of the cerebellar vermis. AAV9 vector suspension (10 μl) was injected at a rate of 300 nl/min using a microprocessor-based controller (Micro4; World Precision Instrument). The syringe was left in place for 2 min following the injection. After closing the scalp, the mice were returned to standard home cages.

For intravenous injection, 100 μl of the PHP.eB vector suspension was injected into the retro-orbital sinus of deeply anesthetized mice using a 0.5-mL syringe with a 30-gauge needle (08277; Nipro, Osaka, Japan).

### 2.4 Electrophysiological experiments

Parasagittal cerebellar slices (250 μm in thickness) from P49–P69 mice were prepared as previously described (Watanave et al., 2018, 2019). The slices were perfused in an extracellular solution containing (in mM): 125 NaCl, 2.5 KCl, 1.25 NaH_2_PO_4_, 26 NaHCO_3_, 2 CaCl_2_, 1 MgCl_2_, 10 glucose, and 0.1 picrotoxin, bubbled continuously with a mixture of 95% O_2_ and 5% CO_2_ at room temperature during the recordings. For some experiments, the extracellular solutions contained 20 μM ZD7288 to block HCN channels. PCs were visualized using a 40× water-immersion objective attached to an upright microscope (Axioskop; Carl Zeiss). The resistance of the patch pipette was 3–6 MΩ when filled with an intracellular solution containing (in mM): 122.5 K-gluconate, 17.5 KCl, 8 NaCl, 2 Mg ATP, 0.3 NaGTP, 10 HEPES, and 0.2 EGTA (pH 7.2, adjusted with KOH, 290-310 Osm). All whole-cell data was obtained using an EPC8 amplifier (HEKA Electronik), controlled with pClamp 10 software (Molecular Device), and analyzed using Clampfit software (Molecular Device). No bridge balance compensation was performed. We discarded the data if the holding current when the cell was held at −70 mV was smaller than −500 pA. The intrinsic properties of PCs were recorded in the current–clamp mode. Negative currents (<−500 pA) were injected to adjust the baseline to approximately −70 mV, and various sizes of depolarizing current steps (500 ms; 50 to 500 pA, in 50 pA increments) were applied to evoke action potentials. The spikes obtained from each current injection were counted. The lowest injected currents which generated action potential were corrected as rheobase currents. The thresholds of the action potentials were obtained at the time point where dV/dt = 30. Input resistances were simultaneously obtained in each recording by applying negative currents (500 ms;−50 pA) to PCs. Inter spike interval (ISI) and adaptation index were obtained from the action potential traces which showed 10–15 action potentials. Adaptation indexes were calculated by dividing 1st ISI by last ISI of each trace. Sag voltage and rebound depolarization were recorded by injecting negative step currents (500 ms; −50 to −250 pA, in 50 pA increments). Sag voltage was calculated by subtracting mean potential at 450–500 ms from the negative peak potentials. Rebound depolarization was measured by subtracting the peak potential of the positive direction at 0–500 ms (after the negative current injection) from baseline potentials. Liquid junction potential was not corrected in this study.

### 2.5 Immunohistochemistry

The mice 4 weeks after the viral injections were deeply anesthetized and transcardially perfused with PBS and 4% PFA (in 0.1 M phosphate buffer), and 50-μm-thick cerebellar slices were obtained. The slices were treated with the following primary antibodies: rat monoclonal anti-GFP (1:1,000; Cat. No. 04404-84, Nacalai Tesque, Kyoto, Japan), rabbit monoclonal anti-PKCγ (1:1,000; Cat. No. AB_2571824; Nittobo Medical, Tokyo, Japan), rabbit polyclonal anti-calbindin 28K (1:500; Cat. No. C2724; Sigma-Aldrich St. Louis, MO, USA), goat polyclonal anti Aldolase-C (1:200; Cat. No. AldolaseC-Go-Af800; Nittobo Medical, Tokyo, Japan) and the following secondary antibodies: Alexa Fluor 488 donkey anti-rat IgG (1:1,000; Thermo Fisher Scientific), Alexa Fluor Plus 488 donkey anti-goat IgG (1:2,000; Thermo Fisher Scientific), Alexa Fluor Plus 555 donkey anti-rabbit IgG (1:2,000; Thermo Fisher Scientific), Alexa Fluor 568 donkey anti-rabbit IgG (1:1,000; Thermo Fisher Scientific). Fluorescence images were acquired using a fluorescence microscope (BZ-X800, Keyence, Osaka, Japan).

### 2.6 Statistical analysis

The sample size of the recording was determined as referring to resembling studies. After we had checked the normality of the data, significant differences were analyzed using Welch’s *t*-test, one-way or two-way repeated measure analysis of variance (ANOVA) followed by Bonferroni’s *post-hoc* test. Statistical analyses were performed using GraphPad Prism 9 (GraphPad Software, San Diego, CA, USA). Data are expressed as the mean ± SEM. The detailed statistics of two-way ANOVA, including F value, degree of free and individual *post-hoc p*-values are compiled in Supplementary Table 1.

## 3 Results

### 3.1 Higher intrinsic excitability in PKCγ-conditional knock-out (PKCγ-cKO) PCs

**FIGURE 1:**
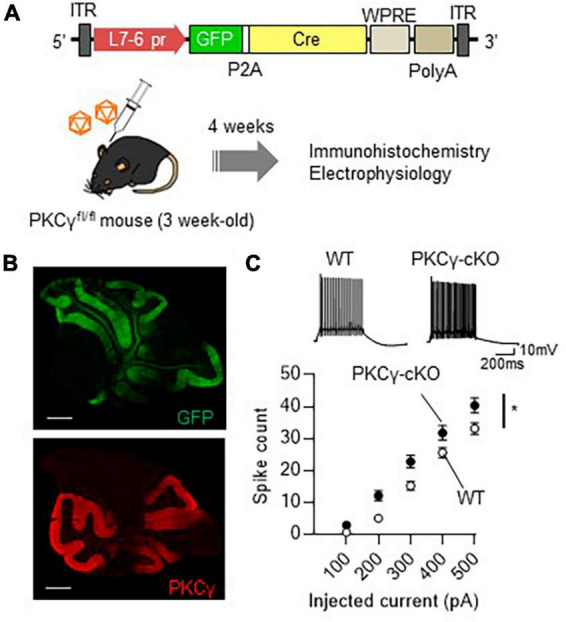
Significant enhancement of intrinsic excitability in protein kinase C γ isoform (PKCγ)-conditional knock-out (cKO) Purkinje cells (PCs). **(A)** Schema depicting the experimental procedure. Three-week-old PKCγ*^fl/fl^* mice and wild-type (WT) mice were administered cerebellar injection of adeno-associated virus serotype 9 (AAV9) vectors expressing Cre recombinase (Cre) together with GFP (GFP-P2A-Cre) under the control of cerebellar PC-specific L7-6 promoter [1.0 × 10^9^ viral genome (vg)/mouse]. ITR, inverted terminal repeat; L7-6 pr, L7-6 promoter with minimal cytomegalovirus sequence; P2A, porcine teschovirus-1 2A self-cleaving peptide; PolyA, polyadenylation signal; WPRE, woodchuck hepatitis virus posttranscriptional regulatory element. **(B)** Immunohistochemistry of the cerebellar section from the AAV-treated PKCγ-cKO mouse. The section was stained with antibodies for GFP (the upper image) and PKCγ (Red, the lower image). **(C)** Enhanced intrinsic excitability in PKCγ-cKO PCs. Intrinsic excitability was examined from PCs randomly chosen from vermal slices, which was assessed by the number of spikes evoked by injection of a somatic depolarizing current (100–500 pA, in 100 pA increments). The representative traces evoked with 300 pA pulses are shown above the graph. Scale bar = 500 μm for **(B)**, 200 ms, 10 mV for **(C)**. *p < 0.05 by two-way repeated measure ANOVA.

Analysis of conventional PKCγ-deficient mice have suggested that PKCγ plays a critical role in brain development (Chen et al., 1995; Kano et al., 1995). Systemic PKCγ-knockout (PKCγ-KO) mice show motor deficits, which may be due to the developmental defects. Therefore, roles of PKCγ in adult mouse PCs have not been fully clarified yet. To assess the physiological significance of PKCγ in mature mouse PCs, we used PKCγ*^fl/fl^* mice that had lost PKCγ expression in a Cre recombinase-dependent manner (Watanave et al., 2022). At 3 weeks of age, the PKCγ*^fl/fl^* mice and their wild-type (WT) littermates were administered a cerebellar injection of AAV9 vectors expressing Cre and GFP under the control of a PC-specific L7-6 promoter (Nitta et al., 2017; Figure 1A). Immunohistochemistry 4 weeks after the AAV injection confirmed conditional knockout of PKCγ specifically in GFP-labeled (Cre-expressing) PKCγ*^fl/fl^* mouse PCs (PKCγ-cKO PCs, hereafter), in contrast with the clear PKCγ immunolabeling in GFP-negative PCs (Figure 1B). To clarify the effect of PKCγ deletion, we examined electrophysiological properties of (GFP-expressing) PKCγ-cKO PCs using acute cerebellar slices of cerebellar vermis. We first recorded the intrinsic excitability of PKCγ-cKO PCs randomly selected from the cerebellar vermis in the current clamp mode. Negative currents were injected to adjust holding potentials to −70 mV, and the spikes were evoked by current injections ranging from 100 to 500 pA in 100 pA increments. PKCγ-cKO PCs showed higher intrinsic excitability than WT mouse PCs (Figure 1C; WT: *n* = 34 from eight mice, PKCγ-cKO: *n* = 33 from seven mice, *p* < 0.01 at 200 pA, *p* < 0.05 at 300 pA by Bonferroni’s *post-hoc* test following 2-way ANOVA). Also, to obtain more detailed intrinsic properties, we have analyzed input resistances (Ri), action potential threshold, and the rheobase currents of those data. Although a significant difference was not detected in Ri and the thresholds between WT and cKO PCs, the rheobase currents were significantly lower in cKO PCs, which could reflect higher intrinsic excitability in cKO PCs (Supplementary Figure 1). These results suggested that PKCγ in mature mouse PCs suppresses the intrinsic excitability of PCs.

### 3.2 Absence of enhanced intrinsic excitability in PKCγ-cKO mouse PCs in lobules IX–X

**FIGURE 2:**
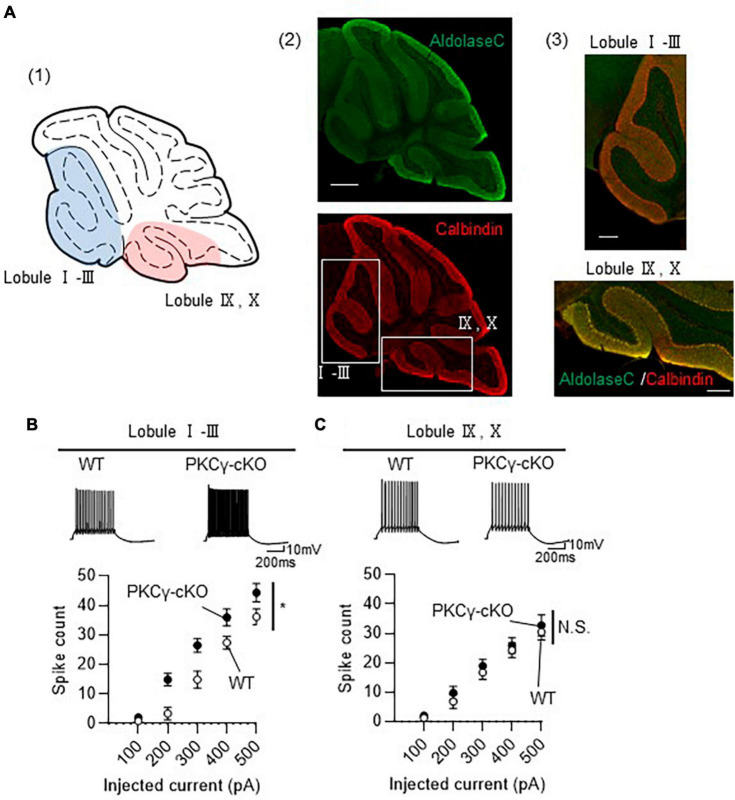
Enhanced intrinsic excitability in protein kinase C γ isoform (PKCγ)-conditional knock-out (cKO) Purkinje cells (PCs) of lobules I–III, but not of lobules IX–X. **(A)** **(A1)** Schema of areas examined for intrinsic excitability. Intrinsic excitability was recorded from PCs localizing in the colored areas of the cerebellar vermis (lobules I–III and lobules IX–X). **(A2,A3)** Immunohistochemistry of the cerebellar vermis section. The section was stained with antibodies for Aldolase C (the upper image) and Calbindin (Red, the lower image). The magnified images of the boxed areas in **(A2)** are shown in **(A3)**. **(B,C)** Graphs showing the change in spike number elicited by gradually increasing injection of current to PCs located in lobules I–III **(B)** and lobules IX–X **(C)**. Traces above graphs show representatives evoked by injection of 300 pA current. Scale bar = 500 μm for **(A2)**, 200 μm for **(A3)**, 200 ms, 10 mV for **(B,C)**. *p < 0.05 by two-way repeated measure ANOVA. N.S., not significant.

Although we randomly chose PCs for electrophysiological analysis in Figure 1, many PCs examined were from middle lobules (IV–VII), which contained both Z− and Z+ PCs, since large portion of GFP-expressing PCs were observed in lobules IV–VII (Figure 1B). To clarify the association of zebrin expression profiles with intrinsic excitability, we recorded the intrinsic excitability of PCs, focusing on lobules I–III and IX–X, containing mostly Z− and Z+ PCs, respectively (Figure 2A). PKCγ-cKO mouse PCs in lobules I–III showed enhanced intrinsic excitability compared with WT mouse PCs (Figure 2B; WT: *n* = 7 from three mice, PKCγ-cKO: *n* = 10 from four mice, *p* < 0.01 at 200 pA, *p <* 0.05 at 300 pA by Bonferroni’s *post-hoc* test following 2-way ANOVA). In contrast, the intrinsic excitability of PCs in lobules IX–X did not differ significantly between genotypes (Figure 2C; WT: *n* = 9 from four mice, PKCγ-cKO: *n* = 7 from five mice, *p* = 0.515 by 2-way ANOVA). Other intrinsic properties are shown in Supplementary Figure 2. Although it is not significant, Ri tends to be lower in cKO PCs than in WT PCs at lobules I–III (Supplementary Figure 2A). The thresholds were comparable between the genotypes at lobules I–III (Supplementary Figure 2B). The rheobase current was significantly lower in cKO PCs than in WT PCs at lobules I–III (Supplementary Figure 2C). No significant differences between WT and cKO PCs were observed at lobules IX–X (Supplementary Figures 2D–F). These results suggested that PKCγ suppresses the intrinsic excitability selectively in lobules I–III.

To investigate whether PKCγ modulates the intrinsic firing patterns, we have analyzed firing patterns of PCs present in lobules I–III. Both WT and PKCγ-cKO PCs exhibited 2 firing patterns, tonic firing and initial burst firing (Supplementary Figure 3A). The ratio of each firing type was almost comparable between WT and PKCγ-cKO PCs (Supplementary Figure 3B), suggesting that PKCγ does not affect the firing pattern at least in PCs of lobules I–III.

### 3.3 Higher intrinsic excitability exclusively in Z− PCs

**FIGURE 3:**
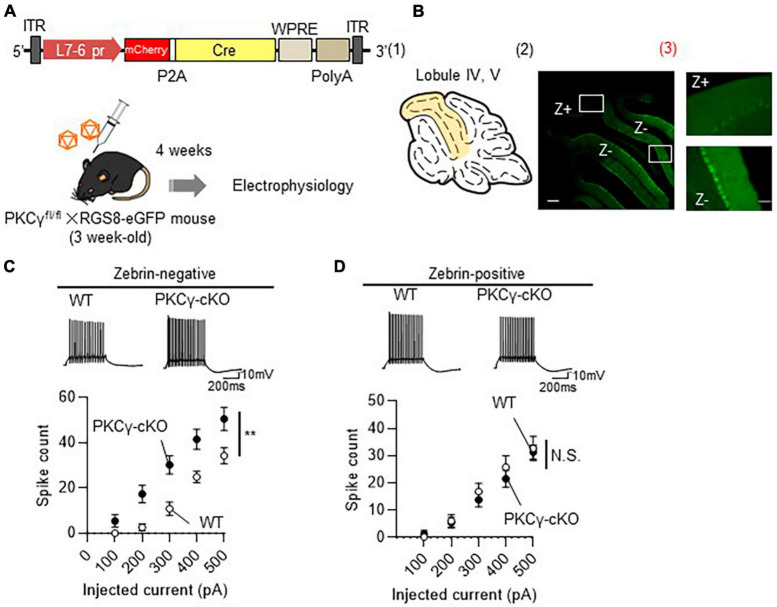
Enhanced intrinsic excitability in zebrin-negative (Z–), but not in zebrin-positive (Z+), Purkinje cells (PCs) of protein kinase C γ isoform (PKCγ)-conditional knock-out (cKO) mice. **(A)** Schema depicting the experimental procedure. PKCγ*^fl/fl^* mice were crossed with RGS8-EGFP mice to obtain mice carrying both genotypes (PKCγ*^fl/fl^* × RGS8-EGFP mouse). Three-week-old PKCγ*^fl/fl^* mice and their wild-type (WT) littermates on RGS8-EGFP background were administered cerebellar injections of adeno-associated virus serotype 9 (AAV9) vectors expressing mCherry-P2A-Cre under the control of cerebellar Purkinje cell-specific L7-6 promoter (1.0 × 10^9^ vg/mouse). Mice treated with AAVs were electrophysiologically analyzed 4 weeks after the viral injection. **(B)** **(B1)** Diagram depicting lobules IV–V (colored area) used for whole cell-recording of PCs. **(B2)** The fluorescent photo on the right shows lobule IV–V containing both PCs positive for GFP (Z–) and negative for GFP (Z+). The magnified images of the boxed areas in **(B2)** are shown in **(B3)**. **(C,D)** Graphs showing the change in spike number elicited by gradually increasing injecting currents to Z– **(C)** and Z+ **(D)** PCs from both AAV-treated WT mice and PKCγ-cKO mice. Traces above graphs show representatives evoked by injection of 300 pA current. Scale bar = 200 μm for (B2), 50 μm for (B3); 200 ms, 10 mV for **(C,D)**. **p < 0.01 by two-way repeated measure ANOVA. N.S., not significant.

To prove that the PKCγ-mediated modulation of intrinsic excitability is present only in Z− PCs, we recorded the intrinsic excitability of Z− and Z+ PCs in the same lobule. To visualize the zebrin expression in PCs, we used RGS8-EGFP mice, that robustly express GFP in Z− PCs (Viet et al., 2022). PKCγ*^fl/fl^* mice were crossed with RGS8-EGFP mice to obtain PKCγ*^fl/fl^*/RGS-EGFP mice. In the following experiments, we used PKCγ*^fl/fl^* mice and their WT littermates on an RGS-EGFP mouse background to differentiate Z− and Z+ PCs. The PKCγ*^fl/fl^* and WT mice (on RGS-EGFP background) were administered cerebellar injections of AAV9 expressing mCherry-P2A-Cre under the PC-specific L7-6 promoter (Figure 3A). The intrinsic excitability was recorded from PCs in lobules IV and V, which contained mixed PCs with strong (Z−) and faint (Z+) GFP fluorescence (Figure 3B and Supplementary Figure 4). Z− PKCγ-deficient PCs (labeled with both GFP and mCherry; PKCγ-cKO) showed significantly enhanced intrinsic excitability compared with Z− WT (PKCγ-expressing) mouse PCs (similarly labeled with both GFP and mCherry) (Figure 3C; WT: *n* = 7 from three mice, PKCγ-cKO: *n* = 9 from four mice, *p* < 0.01 at 300 and 400 pA, *p* < 0.05 at 200 and 500 pA by Bonferroni’s *post-hoc* test following 2-way ANOVA), which was confirmed with lower rheobase in cKO PCs (Supplementary Figure 5). In contrast, the intrinsic excitability for Z+ PCs (labeled clearly with mCherry, but only faintly with GFP) did not differ significantly between PKCγ-deficient mice and control WT mice (Figure 3D; WT: *n* = 11 from five mice, PKCγ-cKO: *n* = 9 from four mice, *p* = 0.634 by 2-way ANOVA).

At this point, to evaluate the effect of PKCγ to the timing of the intrinsic firing, we analyzed inter-spike interval (ISI) and spike adaptation of Z− and Z+ PCs. Although the 1st ISI in Z− PCs are comparable between WT and cKO groups (Supplementary Figure 6A), the adaptation ratio tends to be lower in cKO PCs than in WT PCs (Supplementary Figure 6B). In contrast, both 1st ISI and the adaptation ratio in Z+ PCs are comparable between WT and cKO PCs (Supplementary Figures 6C, D). Although the difference does not reach statistically significant level, PKCγ might affect timing of the intrinsic firing.

**FIGURE 4:**
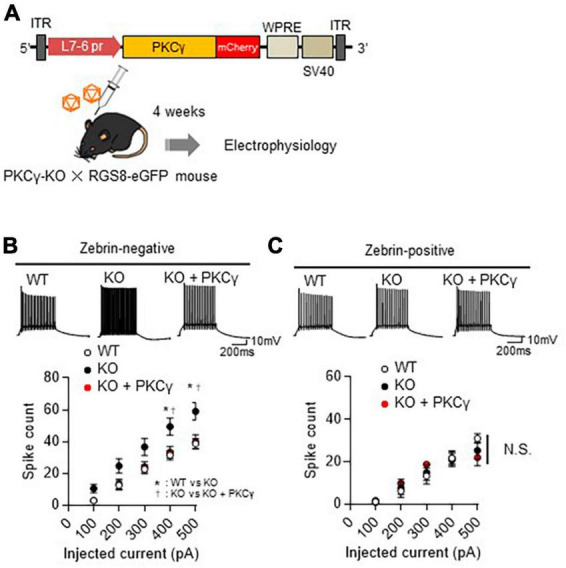
Restoration of enhanced intrinsic excitability in zebrin-negative (Z–) protein kinase C γ isoform (PKCγ)-knock-out (KO) Purkinje cells (PCs) to a level comparable to that of wild-type (WT) mouse PCs by adeno-associated virus (AAV)-mediated re-expression of PKCγ. **(A)** Schema depicting the experimental procedure. Systemic PKCγ-KO mice were crossed with RGS8-EGFP mice to obtain mice carrying both genotypes (PKCγ-KO × RGS8-EGFP). Three-week-old PKCγ-KO mice and their WT littermates on RGS8-EGFP background were intravenously administered the blood-brain barrier-penetrating AAV-PHP.eB vectors expressing PKCγ fused with mCherry under the control of cerebellar PC-specific L7-6 promoter (5 × 10^10^ vector genomes/mouse). Mice treated with AAVs were electrophysiologically analyzed 4 weeks after the viral injection. **(B,C)** Graphs showing the change in spike number elicited by gradually increasing injection of currents to Z– **(B)** and Z+ **(C)** PCs from WT mice, PKCγ-KO mice (KO), and PKCγ-KO mice virally-expressing PKCγ specifically in PCs. Traces above graphs show representatives evoked by injection of 300 pA current. Scale bar = 200 ms, 10 mV. * (WT vs. KO) and † (KO vs. KO + PKCγ) indicate statistically significant (*p* < 0.05 by Bonferroni’s *post-hoc* test following two-way repeated measure ANOVA). N.S., not significant.

To confirm that the intrinsic excitability is regulated by PKCγ exclusively in Z− PCs, we conducted a rescue experiment in which PKCγ was virally restored to PKCγ-KO mouse PCs. Briefly, systemic PKCγ-KO mice (on an RGS-EGFP mouse background) were intravenously injected with blood-brain barrier-penetrating AAV-PHP.eB expressing PKCγ-mCherry under the PC-specific L7-6 promoter (Figure 4A). Four weeks after the viral injection, the intrinsic excitability was recorded from Z− and Z+ PCs labeled with mCherry (KO + PKCγ) in lobules IV and V. Compared with WT mouse PCs, systemic PKCγ-KO mice showed enhanced intrinsic excitability in Z− PCs (Figure 4B; WT: *n* = 8 from three mice, KO: *n* = 12 from three mice, WT vs. KO: *p* < 0.05 at 400 and 500 pA by Bonferroni’s *post-hoc* test following 2-way ANOVA), but not in Z+ PCs (Figure 4C; WT: *n=8* from three mice, KO: *n=7* from three mice, *p* = 0.980 by 2-way ANOVA). AAV-mediated PKCγ expression restored the enhanced intrinsic excitability in Z− PKCγ-KO mouse PCs to a level comparable to that in WT mouse PCs (Figure 4B; KO + PKCγ: *n* = 8 from three mice, KO vs. KO + PKCγ: *p* < 0.05 at 400 and 500 pA by Bonferroni’s *post-hoc* test following 2-way ANOVA). In contrast, re-expression of PKCγ in Z+ PKCγ-KO mouse PCs did not exert any significant influence on the intrinsic excitability (Figure 4C; KO + PKCγ: *n* = 8 from three mice, *p* = 0.980 by 2-way ANOVA). The other intrinsic properties of PCs are shown in Supplementary Figure 7. These results further suggested that PKCγ negatively regulates the intrinsic excitability exclusively in Z− PCs.

### 3.4 Larger HCN channel currents in PKCγ-cKO Z− PCs than in WT Z− PCs

A recent study showed that HCN channel-mediated currents are larger in lobule III PCs (mostly Z− PCs) than in lobule X PCs (mostly Z+ PCs) (Beekhof and Schonewille, 2023). Moreover, HCN currents were shown to be modulated by PKC in cultured mammalian cells (Reetz and Strauss, 2013). Therefore, we examined whether PKCγ could modulate HCN currents, and consequently, contribute to regulation of the intrinsic excitability in Z− PCs.

**FIGURE 5:**
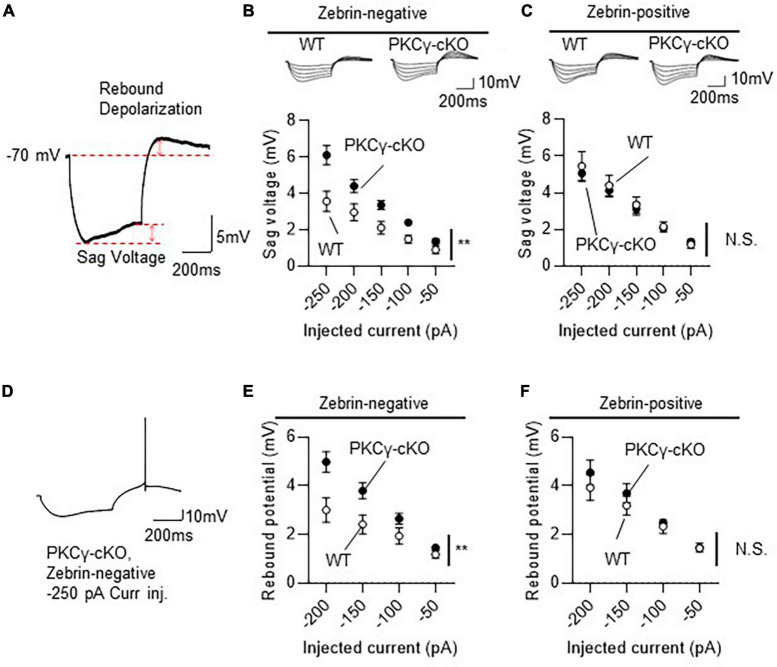
Larger hyperpolarization-activated cyclic nucleotide-gated (HCN) currents in zebrin-negative (Z–), but not zebrin-positive (Z+), protein kinase C γ isoform (PKCγ)-conditional knock-out (cKO) Purkinje cells (PCs). Mice on RGS8-EGFP background were used to distinguish Z– from Z+ PCs. Wild-type (WT) and PKCγ*^fl/fl^* mice received injection of AAV expressing mCherry and Cre under the control of the L7-6 promoter (as described in Figure 3A). The HCN currents were recorded from mCherry expressing Z– PC in lobules IV and V. **(A)** Diagram depicting sag voltage and rebound depolarization during and after injection of hyperpolarizing current to a PC. Sag voltage was defined as the value obtained after subtracting mean potential of the last 50 ms from the negative peak potentials during injection of negative current. Rebound depolarization was defined as the value obtained after subtracting the original membrane potential (–70 mV) from the peak amplitudes after injection of the negative current. **(B)** Graph and representative traces showing significantly larger sag voltage in PKCγ-cKO PCs than in WT PCs in Z– module. Sag voltage was elicited by injecting negative currents varying from –50 to –250 pA, in 50 pA increments. **(C)** No significant difference in sag voltage was elicited in Z+ PCs between PKCγ-cKO and WT mice. **(D–F)** Graphs showing significantly larger rebound depolarization in PKCγ-cKO PCs than in WT mouse PCs only in Z– module **(E,F)**. Some Z– PKCγ-deficient PCs generated action potentials during rebound depolarization after –250 pA current injection **(D)**, therefore, maximum negative current was set to –200 pA. Scale bar = 200 ms, 5 mV for **(A)**, and 200 ms, 10 mV for **(D)**. **p < 0.01 by two-way repeated measure ANOVA. N.S., not significant.

**FIGURE 6:**
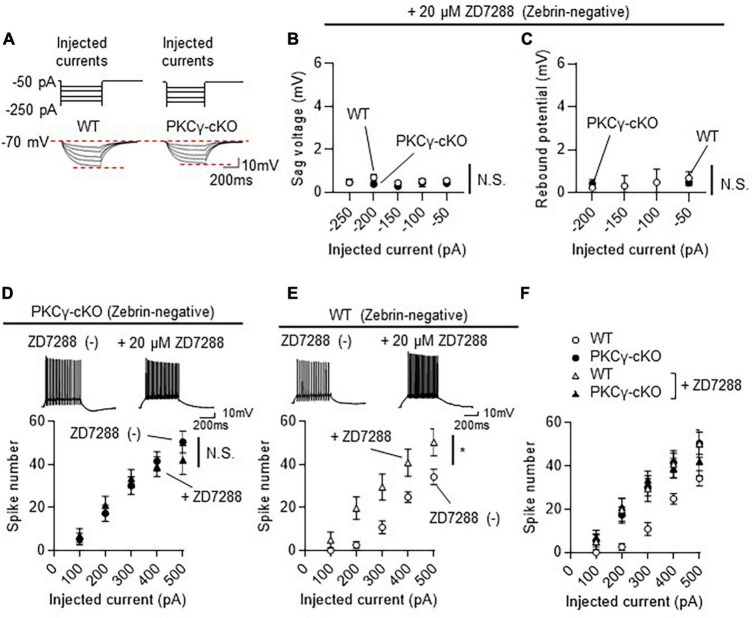
No influence of the inhibition of hyperpolarization-activated cyclic nucleotide-gated (HCN) channels on the intrinsic excitability in protein kinase C γ isoform (PKCγ)-conditional knock-out (cKO) zebrin-negative (Z–) Purkinje cells (PCs). Mice on RGS8-EGFP background were used to distinguish Z– from Z+ PCs. WT and PKCγ*^fl/fl^* mice received injection of AAV expressing mCherry and Cre under the control of the L7-6 promoter (as described in Figure 3A). The HCN currents were recorded from mCherry expressing Z– PC in lobules IV and V. **(A)** Negative currents (–50 to –250 pA, upper traces) were injected to a PC after bath-application of 20 μM ZD7288, an HCN channel blocker. Representative membrane voltage traces are shown (lower traces). **(B,C)** Almost complete elimination of sag voltage **(B)** and rebound potentiation **(C)** in both WT and PKCγ-cKO PCs after ZD7288 application. **(D)** Graph and representative traces showing no significant change in firing frequency after application of 20 μM ZD7288 in PKCγ-cKO PCs. **(E)** Graph and representative traces showing significant increase in firing frequency after application of 20 μM ZD7288 in WT PCs. **(F)** Graph combining **(D,E)**. Scale bars = 200 ms, 10 mV. N.S., not significant, *p < 0.05 by two-way repeated measure ANOVA.

In the subsequent experiments (Figures 5, 6), the HCN currents were recorded in Z+ and Z− PCs in lobules IV and V from mice on RGS8-EGFP background. WT and PKCγ*^fl/fl^* mice that were treated with AAV expressing mCherry and Cre under the control of the L7-6 promoter (as described in Figure 3A). Z+ and Z− PCs were identified by the intensity of GFP fluorescence. The HCN channels were activated by injecting negative currents for 500 ms (−50 to −250 pA, in 50 pA increments) to mCherry-labeled PCs present in lobules IV and V. Because HCN currents, in response to injection of negative currents, generate sag voltage and rebound depolarization in neurons (Han et al., 2017), we compared these voltage changes (in response to injection of negative currents) in WT and PKCγ-cKO mouse PCs, as illustrated in Figure 5A.

Compared to WT mice, PKCγ-cKO mice showed significantly larger sag voltage for Z− PCs (Figure 5B; WT: *n* = 11 from six mice, PKCγ-cKO: *n* = 12 from eight mice, *p* < 0.05 at −100, −150, and −250 pA by Bonferroni’s *post-hoc* test following 2-way ANOVA). In contrast, sizes of sag voltage in Z+ PCs were comparable between PKCγ-cKO and WT mice (Figure 5C; WT: *n* = 11 from eight mice, PKCγ-cKO: *n* = 12 from seven mice, *p* = 0.755 by 2-way ANOVA). Rebound depolarization was calculated by subtracting the baseline potential from the peak amplitude of rebound potentiation following injection of the hyperpolarizing current. Because some of the PKCγ-cKO PCs generated action potentials during the rebound potentiation after injection of −200 to −250 pA current (Figure 5D), rebound depolarization amplitudes were measured from traces obtained by injection of −50 to −200 pA current. Z− PKCγ-cKO PCs showed larger rebound potentiation than Z− WT mouse PCs (Figure 5E; WT: *n* = 11 from seven mice, PKCγ-cKO: *n* = 11 from seven mice, *p* < 0.05 at −150 pA, *p* < 0.01 at −200 pA by Bonferroni’s *post-hoc* test following 2-way ANOVA), whereas the amplitude in Z+ PCs was almost comparable between PKCγ-cKO and WT mice (Figure 5F; WT: *n* = 10 from seven mice, PKCγ-cKO: *n* = 7 from four mice, *p* = 0.456 by 2-way ANOVA). Thus, conditional deletion of PKCγ significantly enhanced sag voltage and rebound potentiation, namely, the HCN currents in Z− PCs, suggesting that PKCγ negatively modulates the HCN currents exclusively in Z− PCs.

### 3.5 PKCγ suppresses the intrinsic excitability in Z− PCs through a mechanism independent of HCN channel regulation

To investigate whether the enhanced HCN currents in PKCγ-cKO PCs in the Z− module underlay the enhanced intrinsic excitability, the HCN channel was blocked by bath-application of 20 μM ZD7288, a blocker of the HCN channels. Both sag voltage and rebound potentiation in Z− PCs of lobules IV and V, which are elicited during and after injection of negative currents, respectively, were almost completely eliminated in the presence of ZD7288 (Figures 6A–C). However, the intrinsic excitability in PKCγ-cKO PCs in the Z− module was not affected by application of ZD7288 (Figure 6D; PKCγ-cKO without ZD7288: *n* = 9 from three mice, PKCγ-cKO with ZD7288: *n* = 9 from three mice, *p* = 0.930 by 2-way ANOVA). The other intrinsic properties of WT and PKCγ-cKO PCs in the presence of ZD7288 are shown in Supplementary Figure 8. These results suggested that enhanced intrinsic excitability in PKCγ-cKO PCs in the Z− module was not due to increase in the HCN currents.

Notably, we found that application of ZD7288 significantly enlarged intrinsic excitability in WT Z− PCs (Figures 6E, F; WT without ZD7288: *n* = 7 from three mice, WT with ZD7288: *n* = 8 from three mice, *p* < 0.05 at 200 and 300 pA by Bonferroni’s *post-hoc* test following 2-way ANOVA) together with the increase in the Ri and decrease in the rheobase current (Supplementary Figure 9).

## 4 Discussion

In this study, using systemic PKCγ-KO and PC-specific PKCγ-cKO mice, we showed that elimination of PKCγ enhances the intrinsic excitability in Z− PCs, but not in Z+ PCs. AAV-mediated re-expression of PKCγ in PKCγ-deficient Z− PCs suppressed the higher intrinsic excitability to a level comparable to that of WT Z− PCs. In parallel with the enhanced intrinsic excitability, PKCγ-deficient Z− PCs showed significantly larger HCN currents, compared with WT Z− PCs. Notably, higher intrinsic excitability in PKCγ-deficient Z− PCs was not restored by the application of ZD7288, a blocker of the HCN channels. These results suggest that PKCγ negatively regulates both the intrinsic excitability and HCN currents solely in Z− PCs; however, those two events are not related each other.

Blockade of HCN channels by ZD7288 enhanced the firing rate in WT Z− PCs (Figure 6E). Similar enhancement of the intrinsic excitability by ZD7288 was reported in entorhinal cortex pyramidal neurons (Huang et al., 2017). Thus, the intrinsic excitability is likely relevant to the HCN channel activity in these neurons. In contrast, it seems that PKCγ suppresses the intrinsic excitability in Z− PCs by a mechanism distinct from regulation of HCN channel currents, because the increased firing rate in PKCγ-cKO Z− PCs was not affected by blockade of HCN currents by ZD7288, which potently inhibited HCN channel activity (Figures 6B, C).

Then, what is a possible mechanism regulating the intrinsic excitability by PKCγ? A possible candidate linking PKCγ and the intrinsic excitability in Z− PCs may be TRPC3. Z− PCs expresses more TRPC3 than Z+ PCs (Wu et al., 2019). PC firing rate in Z−, but not in Z+ PCs, is associated with expression levels of TRPC3; TRPC3 gain-of-function mutant mice (TRPC3 Moonwalker mutant) showed increased firing rate in Z− PCs, whereas targeted deletion of TRPC3 from PCs significantly decreased the firing rate of Z− PCs (Wu et al., 2019). TRPC3 is a substrate of PKCγ, and PKCγ negatively regulates TRPC3 activity in COS-7 cells (Adachi et al., 2008). These results suggest attenuation of TRPC3 activity by PKCγ in PCs, and a resultant decrease in the intrinsic excitability. Conversely, enhanced intrinsic excitability of PKCγ-cKO PCs can be explained by an increase in the TRPC3 activity.

In this study, PKCγ deletion enhanced the intrinsic excitability only in Z− PCs. This can be attributed to the heterogenic expression of molecules associated with PKCγ activation in Z− and Z+ PCs; Z− PCs express PLCβ4, while Z+ PCs express PLCβ3 (Sarna et al., 2006). PLCβ4-KO mice show severe cerebellar phenotypes, similar to those shown by mGluR1-KO mice (Aiba et al., 1994; Kano et al., 1997; Ichise et al., 2000), such as persistent innervation of PCs by multiple climbing fibers, impaired long-term depression at parallel fiber–PC synapses, and severe ataxia (Kim et al., 1997; Kano et al., 1998; Miyata et al., 2001). In contrast, no such cerebellar defects have been reported for PLCβ3-KO mice. Thus, PLCβ3 in Z+ PCs may mediate Gq-coupled mGluR1 signaling, including PKCγ activation, less effectively than that mediated by PLCβ4 in Z− PCs. In addition, unlike Z+ PCs, Z− PCs lack the glutamate transporter EAAT4 (Wadiche and Jahr, 2005), leading to higher glutamate spillover, and consequently, more enhanced activation of mGluR1. Taken together, these results suggest that PKCγ may be activated sufficiently enough to regulate the intrinsic excitability only in Z− PCs. Relevant to the mGluR1-mediated glutamatergic transmission, since patch clamp recordings in this study were made without blockers of glutamatergic transmission, it cannot be excluded the effect of spontaneous glutamatergic inputs to PCs on the intrinsic excitability.

Although further study is required, the present study gives insight into the heterogenic modulation of intrinsic excitability in PCs and provides a new physiological role for PKCγ in adult mouse PCs.

## Data availability statement

The original contributions presented in this study are included in this article/Supplementary material, further inquiries can be directed to the corresponding author.

## Ethics statement

The animal study was approved by the Institutional Committee of Gunma University. The study was conducted in accordance with the local legislation and institutional requirements.

## Author contributions

MW: Conceptualization, Data curation, Formal Analysis, Funding acquisition, Investigation, Project administration, Writing – original draft, Writing – review & editing. MK: Data curation, Investigation, Writing – review & editing. AK: Funding acquisition, Resources, Writing – review & editing. RA: Resources, Writing – review & editing. YF: Resources, Writing – review & editing. YM: Data curation, Writing – review & editing. RK: Resources, Writing – review & editing. HH: Conceptualization, Funding acquisition, Project administration, Supervision, Writing – original draft, Writing – review & editing.

## Supplementary material

Supplementary Figure 1 Intrinsic Properties of WT and PKCγ-cKO PCs randomly recorded from cerebellar vermis. **(A)** Input resistance (WT: 76.61 ± 2.93 MΩ, PKCγ-cKO: 74.90 ± 4.04 MΩ, *p* = 0.733), **(B)** action potential threshold (WT: −45.71 ± 0.74 mV, PKCγ-cKO: −46.55 ± 0.73 mV, *p* = 0.420), and **(C)** rheobase currents (WT: 205.9 ± 11.7 pA, PKCγ-cKO: 153.0 ± 10.0 pA, *p* = 0.0010) were obtained from PCs shown in Figure 1C. N.S., not significant; ***p* < 0.01 by Welch’s *t*-test.

Supplementary Figure 2 Intrinsic Properties of WT and PKCγ-cKO PCs recorded from lobules I-III and IX-X PCs. **(A)** Input resistance (WT: 92.55 ± 8.29 MΩ, PKCγ-cKO: 74.79 ± 6.00 MΩ, *p* = 0.108), **(B)** action potential threshold (WT: −47.83 ± 1.62 mV, PKCγ-cKO: −48.34 ± 0.74 mV, *p* = 0.783) and **(C)** rheobase currents (WT: 214.3 ± 26.1 pA, PKCγ-cKO: 140.0 ± 12.5 pA, *p* = 0.031) were obtained from lobules I-III PCs shown in Figure 2B. Those obtained from lobules IX-X PCs (Figure 2C) are shown in **(D–F)** N.S., not significant; **p* < 0.05 by Welch’s *t*-test.

Supplementary Figure 3 The firing patterns of PCs were overall normal in PKCγ-cKO PCs at lobules I-III. **(A)** Tonic firing PCs and initial burst PCs were seen in lobules I-III. The representative traces obtained with positive current injection of 250 (upper traces) and 500 pA (lower traces) are shown. **(B)** Almost two-thirds of WT and PKCγ-cKO PCs in lobules I-III showed tonic firing, while the others showed an initial burst.

Supplementary Figure 4 Cre expression profile in cerebellar sections visualized by co-expressed mCherry. WT × RGS8-EGFP and PKC*^fl/fl^* × RGS8- EGFP mice received cerebellar injection of AAV9 vectors expressing mCherry-P2A-Cre under the control of L7-6 promoter, and the cerebellar sections were produced 4 weeks after the viral injection. **(A)** Low- magnified fluorescent images of native GFP (left) and native mCherry (middle) from whole cerebellar sections and the overlayed images (right). **(B)** Enlarged fluorescent GFP and mCherry images of lobules IV-V and the merged images. Scale bar = 1 mm for **(A)**, and 200 μm for **(B)**.

Supplementary Figure 5 Intrinsic Properties of WT and PKCγ-cKO PCs recorded from Z− and Z+ PCs in lobule IV, V. **(A)** Input resistance (WT: 79.97 ± 4.83 MΩ, PKCγ-cKO: 65.33 ± 6.07 MΩ, *p* = 0.080), **(B)** action potential threshold (WT: −47.84 ± 1.54 mV, PKCγ-cKO: −48.08 ± 1.05 mV, *p* = 0.899), and **(C)** rheobase currents (WT: 235.7 ± 32.2 pA, PKCγ-cKO: 133.3 ± 22.1 pA, *p* = 0.024) were obtained from Z− PCs of lobules IV, V shown in Figure 3C. Those obtained from Z+ PCs (Figure 3D) are shown in **(D–F)** N.S., not significant; **p* < 0.05 by Welch’s *t*-test.

Supplementary Figure 6 Inter-spike interval and spike adaptation of WT and PKCγ-cKO Z− and Z+ PCs. 1st ISI and adaptation index were obtained from traces show 10–15 action potential during 500 ms positive current injection from −70 mV. The 1st ISI is the interval of first two spikes. The adaptation index was calculated by dividing 1st ISI with ISI of last two spikes. 1st ISI and adaptation index of WT and cKO Z− PCs are shown in (**A**; WT: *n* = 6 from three mice, 27.5 ± 3.3 ms, PKCγ-cKO: *n* = 7 from 3 mice, 27.9 ± 1.9 ms, *p* = 0.917) and (**B**; WT: *n* = 6 from three mice, 0.846 ± 0.131, PKCγ-cKO: *n* = 7 from 3 mice, 0.577 ± 0.046, *p* = 0.917). Those of Z+ PCs are shown in (**C**; WT: *n* = 6 from three mice, 30.7 ± 3.1 ms, PKCγ-cKO: *n* = 7 from 3 mice, 29.8 ± 3.3 ms, *p* = 0.844) and (**D**; WT: *n* = 6 from three mice, 1.191 ± 0.286, PKCγ-cKO: *n* = 7 from 3 mice, 1.065 ± 0.156, *p* = 0.700). N.S., not significant by Welch’s *t*-test.

Supplementary Figure 7 Intrinsic Properties of WT, KO and KO + PKCγ PCs recorded from Z− and Z+ PCs at lobule IV, V. **(A)** Input resistance (WT: 88.30 ± 11.2 MΩ, KO: 67.54 ± 5.96 MΩ, KO + PKCγ: 94.38 ± 7.34 MΩ, *p* = 0.078 in One-way ANOVA), **(B)** action potential threshold (WT: −49.62 ± 1.02 mV, KO: −51.53 ± 1.33 mV, KO + PKCγ: −48.32 ± 1.84 mV, *p* = 0.276 in One-way ANOVA), and **(C)** rheobase currents (WT: 144.4 ± 21.2 pA, KO: 120.8 ± 22.6 pA, KO + PKCγ: 150.0 ± 18.9 pA, *p* = 0.541 in One-way ANOVA) were obtained from Z− PCs of lobules IV, V shown in Figure 4B. Those obtained from Z+ PCs (Figure 4C) are shown in **(D–F)** N.S., not significant by One-way ANOVA.

Supplementary Figure 8 Intrinsic properties of WT and PKCγ-cKO PCs recorded from Z−and Z+ PCs at lobule IV, V in the presence of 20 μM ZD7288. **(A)** Input resistance (WT: 107.3 ± 10.3 MΩ, PKCγ-cKO: 121.2 ± 13.3 MΩ, *p* = 0.422), **(B)** action potential threshold (WT: −47.37 ± 1.32 mV, PKCγ-cKO: −49.59 ± 0.90 mV, *p* = 0.190), and **(C)** rheobase currents (WT: 131.3 ± 16.2 pA, PKCγ-cKO: 122.2 ± 14.7 pA, *p* = 0.686) were obtained in presence of 20 μM ZD7288 in extracellular solution from Z− PCs of lobules IV, V shown in Figure 6B. N.S., not significant by Welch’s *t*-test.

Supplementary Figure 9 Increase in the input resistance and decrease in the rheobase currents after application of 20 μM ZD7288. **(A)** Input resistance (*p* = 0.037), **(B)** action potential threshold (*p* = 0.819), and **(C)** rheobase currents (*p* = 0.018) were compared in absence (same data as shown in WT of Supplementary Figures 5A–C) and presence (same data as shown in WT of Supplementary Figures 8A–C) of 20 μM ZD7288. N.S., not significant; **p* < 0.05 by Welch’s *t*-test.
