# Supplementary material for: Protein kinase Cγ negatively regulates the intrinsic excitability in zebrin-negative cerebellar Purkinje cells
Source: Front Cell Neurosci. 2024 Feb 16;18:1349878. doi: 10.3389/fncel.2024.1349878 (PMC10904455; doi:10.3389/fncel.2024.1349878)
Supplement: Supplementary file 1 [file Table_1.DOCX]

| **Figure 1C** | F (1, 65) = 8.962 | | p=0.004 | by Two-Way ANOVA | |  |
| --- | --- | --- | --- | --- | --- | --- |
| WT vs cKO | 100pA | p=0.125 | 200pA | p=0.003 | 300pA | p=0.012 |
|  | 400pA | p=0.145 | 500pA | p=0.101 | by Bonferroni’s Post hoc | |
| **Figure 2B** | F (1, 15) = 7.116 | | p=0.018 | by Two-Way ANOVA | |  |
| WT vs cKO | 100pA | p>0.999 | 200pA | p=0.010 | 300pA | p=0.041 |
|  | 400pA | p=0.155 | 500pA | p=0.335 | by Bonferroni’s Post hoc | |
| **Figure 2C** | F (1, 14) = 0.445 | | p=0.515 | by Two-Way ANOVA | |  |
| **Figure 3C** | F (1, 14) = 9.660 | | p=0.008 | by Two-Way ANOVA | |  |
| WT vs cKO | 100pA | p>0.999 | 200pA | p=0.028 | 300pA | p=0.002 |
|  | 400pA | p=0.009 | 500pA | p=0.012 | by Bonferroni’s Post hoc | |
| **Figure 3D** | F (1, 18) = 0.234 | | p=0.634 | by Two-Way ANOVA | |  |
| **Figure 4B** | F (2, 25) = 4.418 | | p=0.023 | by Two-Way ANOVA | |  |
| WT vs KO | 100pA | p=0.056 | 200pA | p=0.073 | 300pA | p=0.075 |
|  | 400pA | p=0.019 | 500pA | p=0.012 |  | |
| KO vs KO+PKCγ | 100pA | p=0.055 | 200pA | p=0.102 | 300pA | p=0.125 |
|  | 400pA | p=0.046 | 500pA | p=0.030 | by Bonferroni’s Post hoc | |
| **Figure 4C** | F (2, 20) =0.0207 | | p=0.980 | by Two-Way ANOVA | |  |
| **Figure 5B** | F (1, 21) = 9.987 | | p=0.005 | by Two-Way ANOVA | |  |
| WT vs cKO | -50pA | p=0.510 | -100pA | p=0.022 | -150pA | p=0.041 |
|  | -200pA | p=0.115 | -250pA | p=0.018 | by Bonferroni’s Post hoc | |
| **Figure 5C** | F (1, 21) =0.1004 | | p=0.755 | by Two-Way ANOVA | |  |
| **Figure 5E** | F (1, 20) =9.857 | | p=0.005 | by Two-Way ANOVA | |  |
| WT vs cKO | -50pA | p=0.232 | -100pA | p=0.111 |  |  |
|  | -150pA | p=0.016 | -200pA | p=0.009 | by Bonferroni’s Post hoc | |
| **Figure 5F** | F (1, 15) = 0.587 | | p=0.456 | by Two-Way ANOVA | |  |
| **Figure 6B** | F (1, 12) = 0.726 | | p=0.411 | by Two-Way ANOVA | |  |
| **Figure 6C** | F (1, 12) = 0.489 | | p=0.498 | by Two-Way ANOVA | |  |
| **Figure 6D** | F (1, 16) = 0.0079 | | p=0.930 | by Two-Way ANOVA | |  |
| **Figure 6E** | F (1, 13) = 6.009 | | p=0.029 | by Two-Way ANOVA | |  |
| ZD- vs ZD+ | 100pA | p>0.999 | 200pA | p=0.047 | 300pA | p=0.024 |
|  | 400pA | p=0.076 | 500pA | p=0.074 | by Bonferroni’s Post hoc | |

**Supplementary Table 1**
