# Supplementary figures and images for: Protein kinase Cγ negatively regulates the intrinsic excitability in zebrin-negative cerebellar Purkinje cells
Source: Front Cell Neurosci. 2024 Feb 16;18:1349878. doi: 10.3389/fncel.2024.1349878 (PMC10904455; doi:10.3389/fncel.2024.1349878)

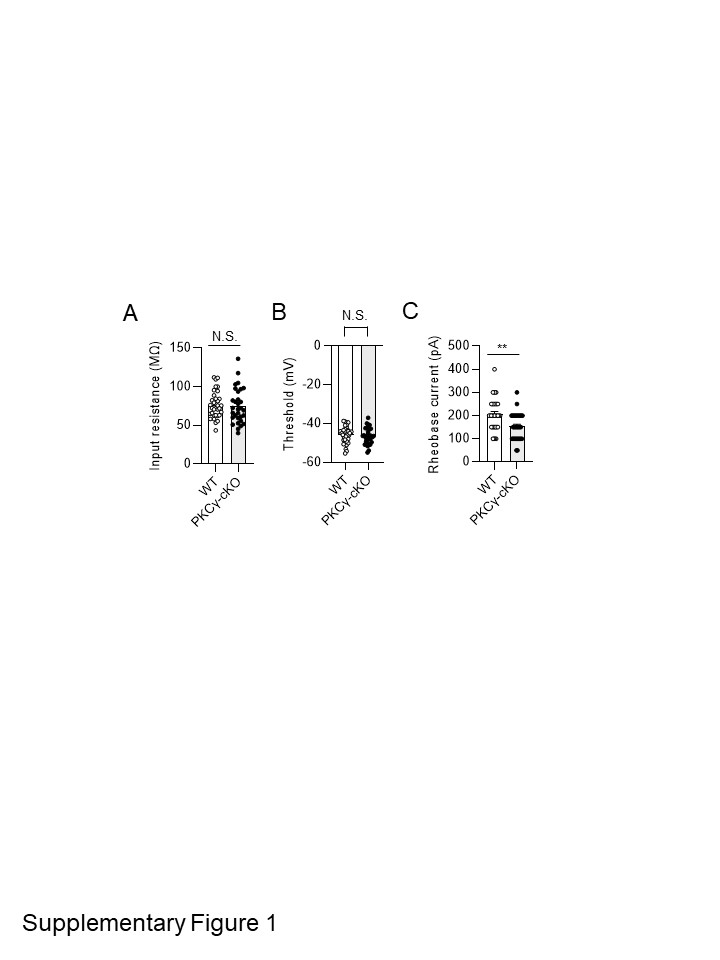

Supplement: Supplementary Figure 1 — Intrinsic Properties of WT and PKCγ-cKO PCs randomly recorded from cerebellar vermis. (A) Input resistance (WT: 76.61 ± 2.93 MΩ, PKCγ-cKO: 74.90 ± 4.04 MΩ, p = 0.733), (B) action potential threshold (WT: −45.71 ± 0.74 mV, PKCγ-cKO: −46.55 ± 0.73 mV, p = 0.420), and (C) rheobase currents (WT: 205.9 ± 11.7 pA, PKCγ-cKO: 153.0 ± 10.0 pA, p = 0.0010) were obtained from PCs shown in Figure 1C. N.S., not significant; **p < 0.01 by Welch’s t-test. [file Image_1.jpg]

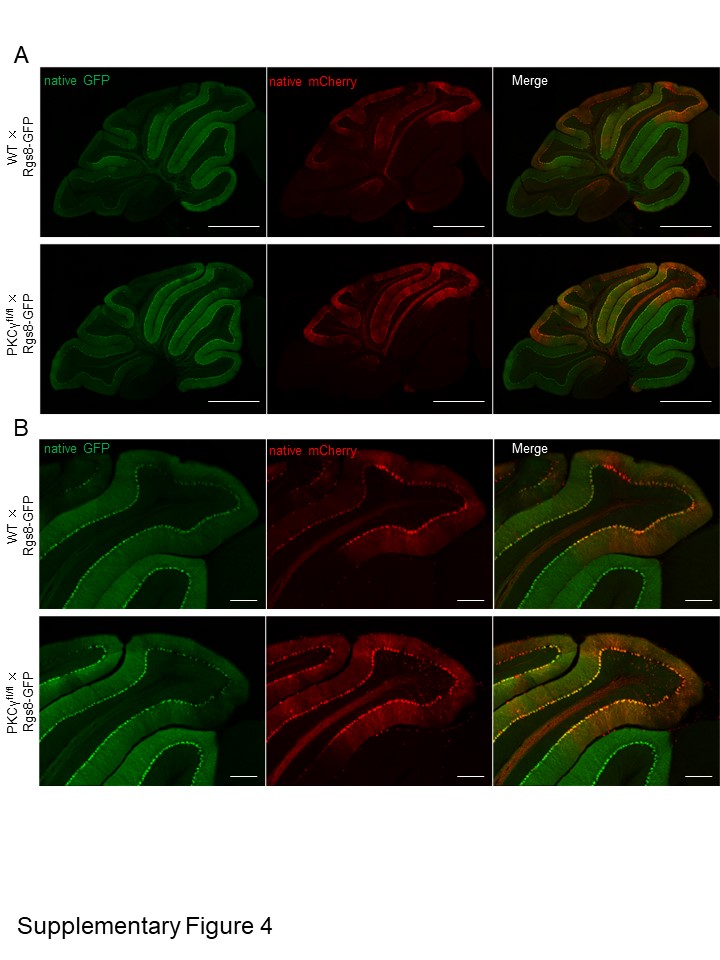

Supplement: Supplementary Figure 2 — Intrinsic Properties of WT and PKCγ-cKO PCs recorded from lobules I-III and IX-X PCs. (A) Input resistance (WT: 92.55 ± 8.29 MΩ, PKCγ-cKO: 74.79 ± 6.00 MΩ, p = 0.108), (B) action potential threshold (WT: −47.83 ± 1.62 mV, PKCγ-cKO: −48.34 ± 0.74 mV, p = 0.783) and (C) rheobase currents (WT: 214.3 ± 26.1 pA, PKCγ-cKO: 140.0 ± 12.5 pA, p = 0.031) were obtained from lobules I-III PCs shown in Figure 2B. Those obtained from lobules IX-X PCs (Figure 2C) are shown in (D–F) N.S., not significant; *p < 0.05 by Welch’s t-test. [file Image_2.jpg]

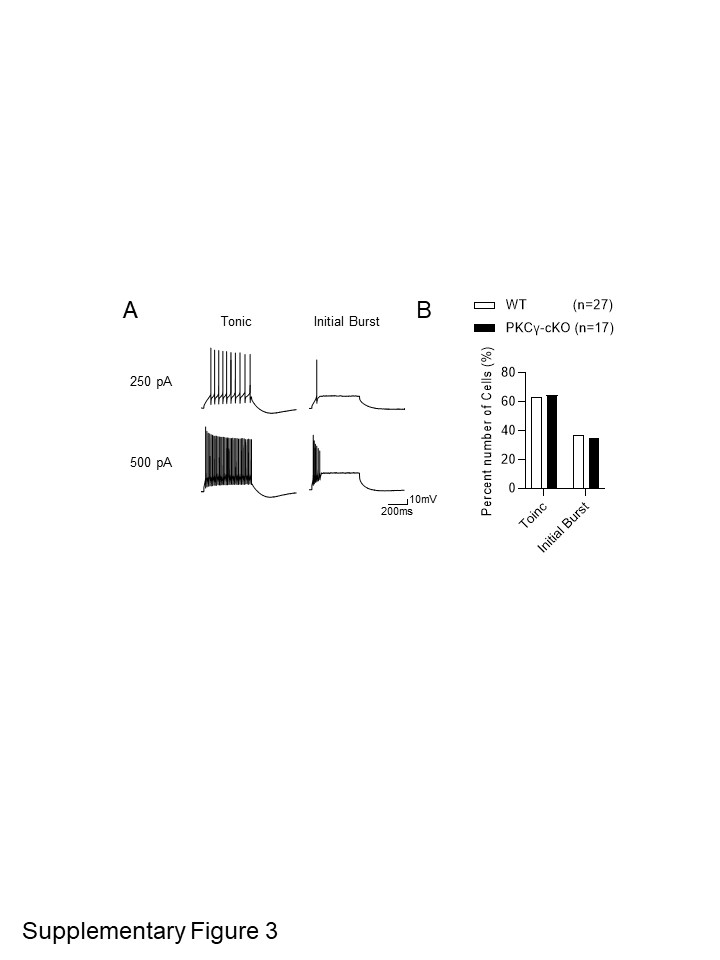

Supplement: Supplementary Figure 3 — The firing patterns of PCs were overall normal in PKCγ-cKO PCs at lobules I-III. (A) Tonic firing PCs and initial burst PCs were seen in lobules I-III. The representative traces obtained with positive current injection of 250 (upper traces) and 500 pA (lower traces) are shown. (B) Almost two-thirds of WT and PKCγ-cKO PCs in lobules I-III showed tonic firing, while the others showed an initial burst. [file Image_3.JPEG]

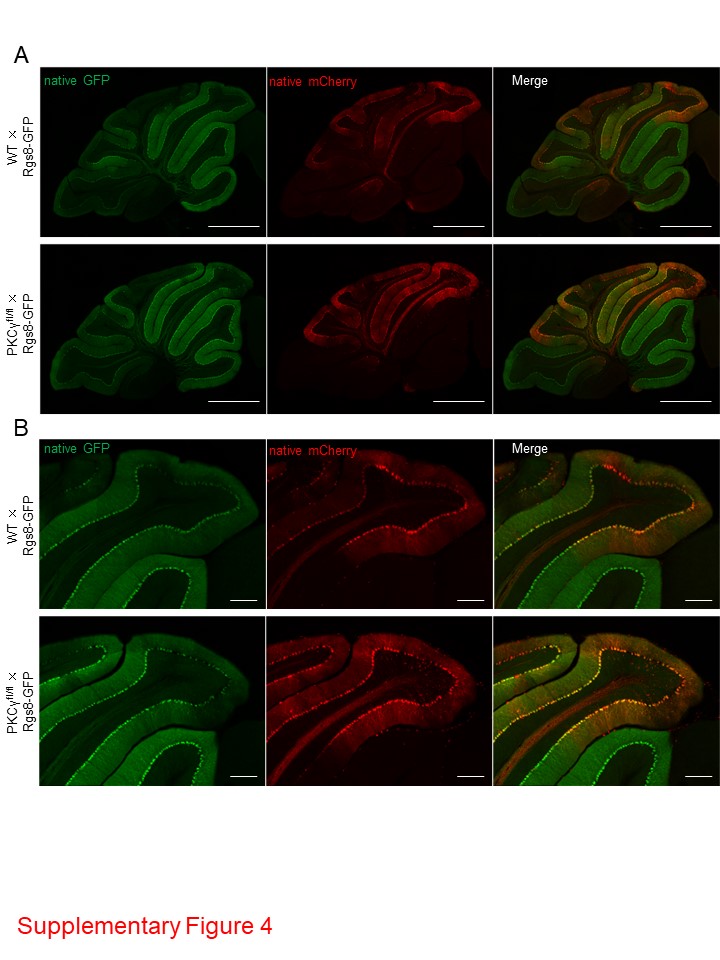

Supplement: Supplementary Figure 4 — Cre expression profile in cerebellar sections visualized by co-expressed mCherry. WT × RGS8-EGFP and PKCfl/fl × RGS8- EGFP mice received cerebellar injection of AAV9 vectors expressing mCherry-P2A-Cre under the control of L7-6 promoter, and the cerebellar sections were produced 4 weeks after the viral injection. (A) Low- magnified fluorescent images of native GFP (left) and native mCherry (middle) from whole cerebellar sections and the overlayed images (right). (B) Enlarged fluorescent GFP and mCherry images of lobules IV-V and the merged images. Scale bar = 1 mm for (A), and 200 μm for (B). [file Image_4.JPEG]

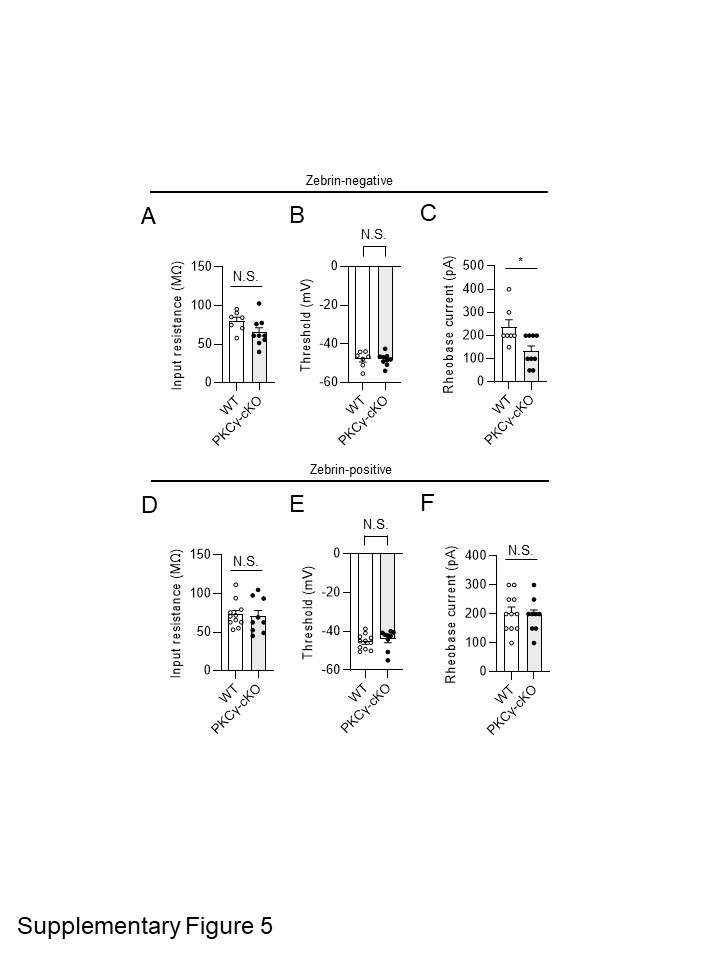

Supplement: Supplementary Figure 5 — Intrinsic Properties of WT and PKCγ-cKO PCs recorded from Z− and Z+ PCs in lobule IV, V. (A) Input resistance (WT: 79.97 ± 4.83 MΩ, PKCγ-cKO: 65.33 ± 6.07 MΩ, p = 0.080), (B) action potential threshold (WT: −47.84 ± 1.54 mV, PKCγ-cKO: −48.08 ± 1.05 mV, p = 0.899), and (C) rheobase currents (WT: 235.7 ± 32.2 pA, PKCγ-cKO: 133.3 ± 22.1 pA, p = 0.024) were obtained from Z− PCs of lobules IV, V shown in Figure 3C. Those obtained from Z+ PCs (Figure 3D) are shown in (D–F) N.S., not significant; *p < 0.05 by Welch’s t-test. [file Image_5.JPEG]

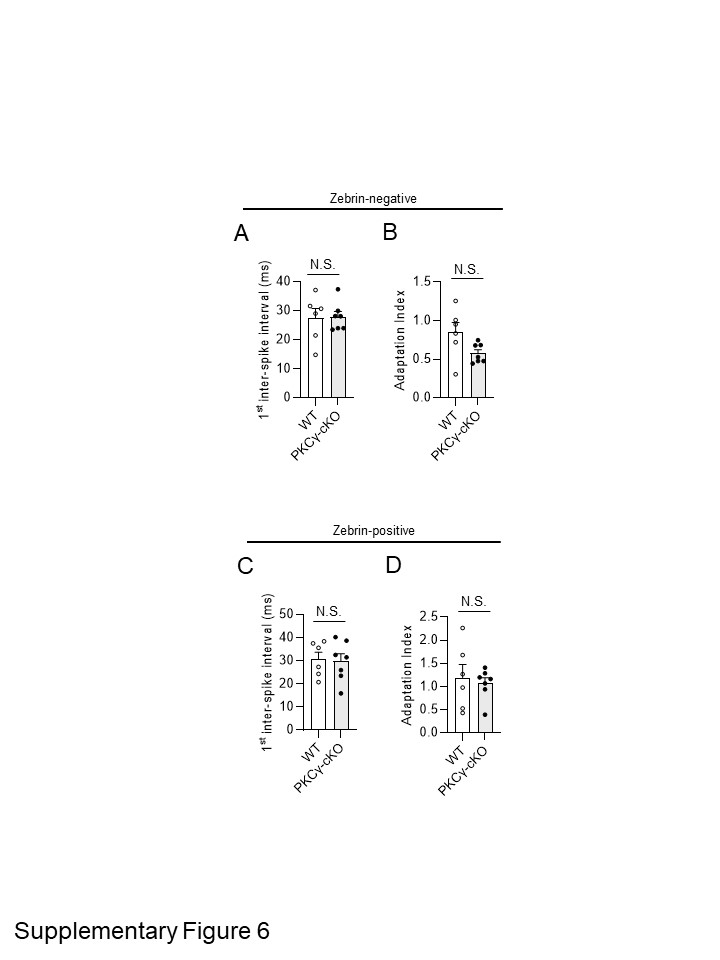

Supplement: Supplementary Figure 6 — Inter-spike interval and spike adaptation of WT and PKCγ-cKO Z− and Z+ PCs. 1st ISI and adaptation index were obtained from traces show 10–15 action potential during 500 ms positive current injection from −70 mV. The 1st ISI is the interval of first two spikes. The adaptation index was calculated by dividing 1st ISI with ISI of last two spikes. 1st ISI and adaptation index of WT and cKO Z− PCs are shown in (A; WT: n = 6 from three mice, 27.5 ± 3.3 ms, PKCγ-cKO: n = 7 from 3 mice, 27.9 ± 1.9 ms, p = 0.917) and (B; WT: n = 6 from three mice, 0.846 ± 0.131, PKCγ-cKO: n = 7 from 3 mice, 0.577 ± 0.046, p = 0.917). Those of Z+ PCs are shown in (C; WT: n = 6 from three mice, 30.7 ± 3.1 ms, PKCγ-cKO: n = 7 from 3 mice, 29.8 ± 3.3 ms, p = 0.844) and (D; WT: n = 6 from three mice, 1.191 ± 0.286, PKCγ-cKO: n = 7 from 3 mice, 1.065 ± 0.156, p = 0.700). N.S., not significant by Welch’s t-test. [file Image_6.JPEG]

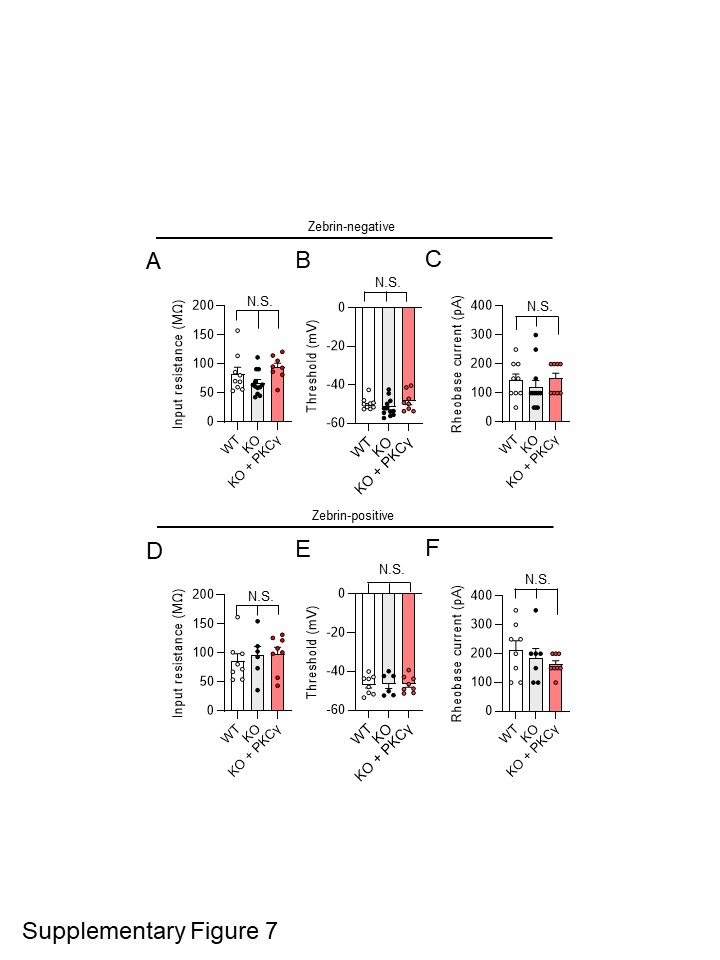

Supplement: Supplementary Figure 7 — Intrinsic Properties of WT, KO and KO + PKCγ PCs recorded from Z− and Z+ PCs at lobule IV, V. (A) Input resistance (WT: 88.30 ± 11.2 MΩ, KO: 67.54 ± 5.96 MΩ, KO + PKCγ: 94.38 ± 7.34 MΩ, p = 0.078 in One-way ANOVA), (B) action potential threshold (WT: −49.62 ± 1.02 mV, KO: −51.53 ± 1.33 mV, KO + PKCγ: −48.32 ± 1.84 mV, p = 0.276 in One-way ANOVA), and (C) rheobase currents (WT: 144.4 ± 21.2 pA, KO: 120.8 ± 22.6 pA, KO + PKCγ: 150.0 ± 18.9 pA, p = 0.541 in One-way ANOVA) were obtained from Z− PCs of lobules IV, V shown in Figure 4B. Those obtained from Z+ PCs (Figure 4C) are shown in (D–F) N.S., not significant by One-way ANOVA. [file Image_7.JPEG]

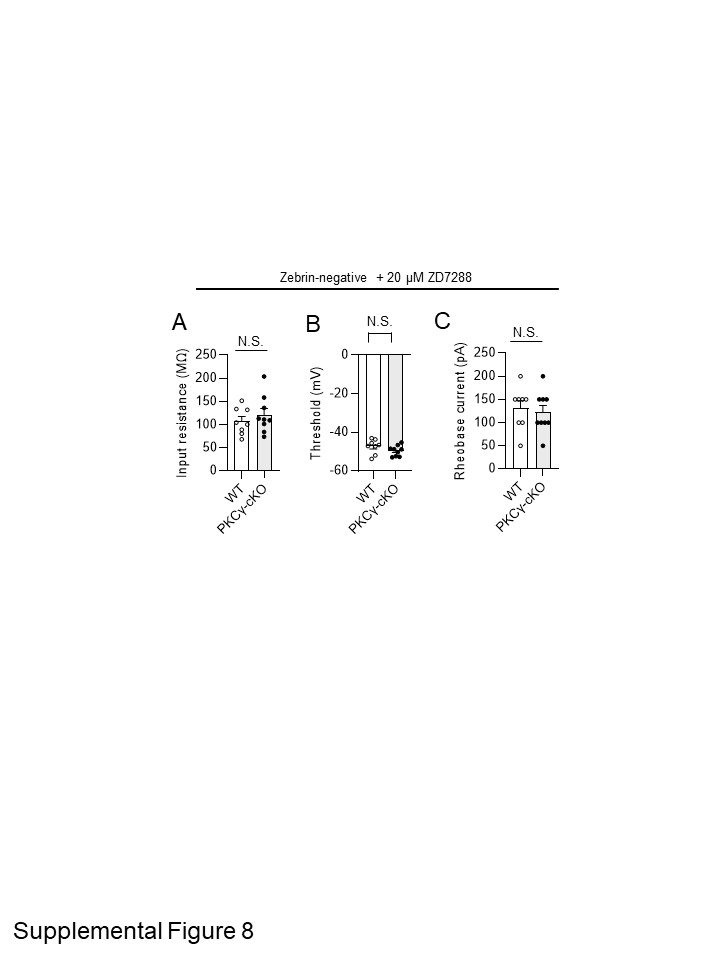

Supplement: Supplementary Figure 8 — Intrinsic properties of WT and PKCγ-cKO PCs recorded from Z−and Z+ PCs at lobule IV, V in the presence of 20 μM ZD7288. (A) Input resistance (WT: 107.3 ± 10.3 MΩ, PKCγ-cKO: 121.2 ± 13.3 MΩ, p = 0.422), (B) action potential threshold (WT: −47.37 ± 1.32 mV, PKCγ-cKO: −49.59 ± 0.90 mV, p = 0.190), and (C) rheobase currents (WT: 131.3 ± 16.2 pA, PKCγ-cKO: 122.2 ± 14.7 pA, p = 0.686) were obtained in presence of 20 μM ZD7288 in extracellular solution from Z− PCs of lobules IV, V shown in Figure 6B. N.S., not significant by Welch’s t-test. [file Image_8.JPEG]

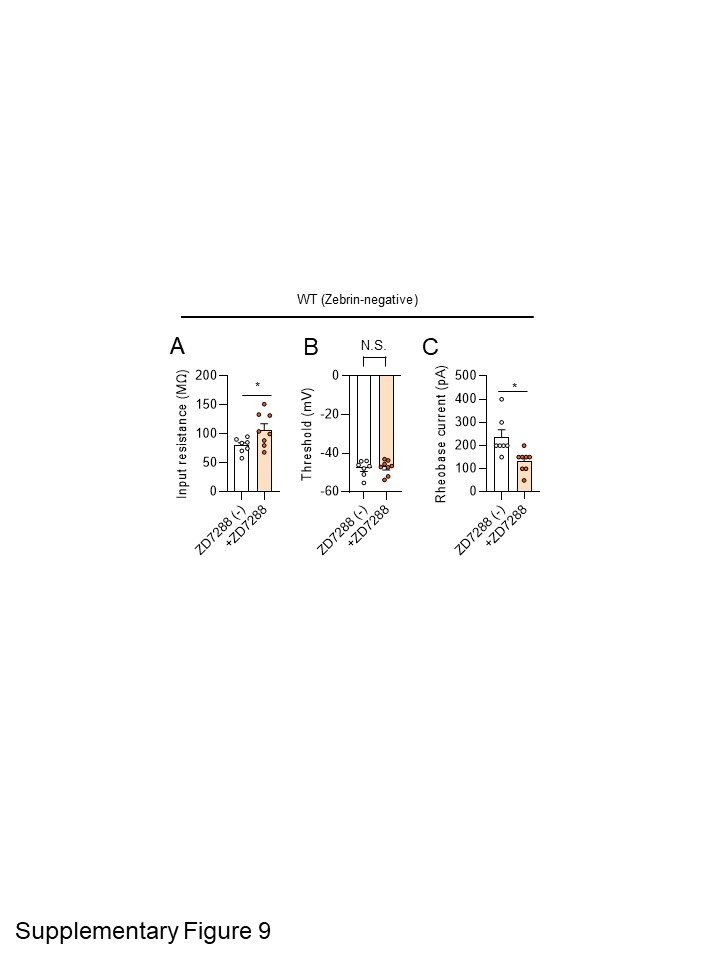

Supplement: Supplementary Figure 9 — Increase in the input resistance and decrease in the rheobase currents after application of 20 μM ZD7288. (A) Input resistance (p = 0.037), (B) action potential threshold (p = 0.819), and (C) rheobase currents (p = 0.018) were compared in absence (same data as shown in WT of Supplementary Figures 5A–C) and presence (same data as shown in WT of Supplementary Figures 8A–C) of 20 μM ZD7288. N.S., not significant; *p < 0.05 by Welch’s t-test. [file Image_9.JPEG]
